# Supplementary material for: Sugar-Sweetened Beverages, Foods of Low Nutritional Value, and Child Undernutrition in Cambodia
Source: Int J Environ Res Public Health. 2024 Feb 1;21(2):169. doi: 10.3390/ijerph21020169 (PMC10887798; doi:10.3390/ijerph21020169)
Supplement: Supplementary file 1 [file ijerph-21-00169-s001.zip › Supplemenatry Tables S1-S2.pdf]

Table S1. Association between flavored sugary drinks and foods of low nutritional value consumed on the previous day and stunting at 24 months stratified by original Cambodia SMILE study group assignment†

	Intervention Group			Control Group		
**Exposure Variable**	**OR**	**[95% CI]**	***p*-value**	**OR**	**[95% CI]**	***p*-value**
Any flavored sugary drinks‡	1.12	[0.54-2.35]	0.76	4.03	[1.79-9.07]	0.001
Any FLNV‡	0.78	[0.39-1.60]	0.50	1.18	[0.45-3.10]	0.74
Number of FLNV on previous day						
1	1.11	[0.48-2.56]	0.80	1.11	[0.37-3.36]	0.85
2	1.04	[0.40-2.70]	0.94	0.96	[0.32-2.92]	0.95
3 or more	0.34	[0.13-0.90]	0.03	1.37	[0.45-4.11]	0.58
Any packaged salty snacks	0.50	[0.26-0.98]	0.04	0.91	[0.46-1.83]	0.80
Number of packaged salty snacks on previous day						
1	0.56	[0.27-1.16]	0.12	0.93	[0.43-2.00]	0.85
2 or more	0.37	[0.14-0.96]	0.04	0.88	[0.34-2.23]	0.78
Any packaged sweets	0.65	[0.32-1.32]	0.23	1.58	[0.77-3.24]	0.21
Number of packaged sweets on previous day						
1	0.91	[0.37-2.23]	0.83	1.76	[0.72-4.31]	0.22
2 or more	0.42	[0.15-1.17]	0.10	1.40	[0.54-3.60]	0.49

†Odds ratios generated using multivariate logistic regression; adjusted for child sex, household income, mother's occupation; N = 175 (Intervention Group), N = 155-157 (Control Group)

‡Flavored sugary drinks include fruit juice, soda, and syrups; Foods of Low Nutritional Value (FLNV) include packaged salty snacks, packaged sweets, and Khmer sweet cake

Table S2. Association between flavored sugary drinks and foods of low nutritional value consumed on the previous day and wasting at 24 months stratified by original Cambodia SMILE study group assignment†

	Intervention Group			Control Group		
**Exposure Variable**	**OR**	**[95% CI]**	***p*-value**	**OR**	**[95% CI]**	***p*-value**
Any flavored sugary drinks‡	2.39	[1.13-5.04]	0.02	1.89	[0.65-5.54]	0.25
Any FLNV‡	1.98	[0.85-4.62]	0.12	1.21	[0.26-5.66]	0.81
Number of FLNV on previous day						
1	1.70	[0.64-4.49]	0.29	1.76	[0.33-9.40]	0.51
2	3.40	[1.23-9.39]	0.02	1.43	[0.26-7.94]	0.68
3 or more	1.34	[0.44-4.09]	0.61	0.67	[0.10-4.25]	0.67
Any packaged salty snacks	2.30	[1.07-4.95]	0.03	0.45	[0.16-1.28]	0.14
Number of packaged salty snacks on previous day						
1	2.84	[1.27-6.33]	0.01	0.30	[0.08-1.17]	0.08
2 or more	1.31	[0.44-3.91]	0.63	0.72	[0.21-2.51]	0.61
Any packaged sweets	1.18	[0.56-2.51]	0.66	1.39	[0.49-3.94]	0.53
Number of packaged sweets on previous day						
1	2.02	[0.84-4.87]	0.12	2.57	[0.84-7.84]	0.10
2 or more	0.50	[0.14-1.78]	0.28	0.37	[0.05-3.06]	0.36

†Odds ratios generated using multivariate logistic regression; not adjusted; N = 183 (Intervention Group), N = 177-179 (Control Group)

‡Flavored sugary drinks include fruit juice, soda, and syrups; Foods of Low Nutritional Value (FLNV) include packaged salty snacks, packaged sweets, and Khmer sweet cake
