# Supplementary material for: Sugar-Sweetened Beverages, Foods of Low Nutritional Value, and Child Undernutrition in Cambodia
Source: Int J Environ Res Public Health. 2024 Feb 1;21(2):169. doi: 10.3390/ijerph21020169 (PMC10887798; doi:10.3390/ijerph21020169)
Supplement: Supplementary file 1 [file ijerph-21-00169-s001.zip › Supplementary File S1.pdf]

2 year evaluation form (control and intervention groups)

General information Date of interview:

Name of Health Centre:		ID number:
Name of provider (nurse/midwife):		ID number:
Name of baby:		ID number:
Date of birth (baby):		Sex: Male ...... Female ......
Mother's name:	Age:	Phone number:
Father's name:	Age:	Phone number:
Name of grandmother:		Phone number:
Name of chief of village:		Phone number:

The first questions relate to self-reported oral health of the mother

Did you brush your teeth yesterday?			Yes	No
Did you use toothpaste with fluoride?			Yes	No
Please rate your oral health :	Poor			Excellent
How much do your mouth and teeth bother you?	No problem			Bother me all the time
How often have you experienced problems with your mouth, teeth or jaws in the last month?	Never	Once or twice	Every week	Every day or almost everyday

Socioeconomic description of the mother

What is your highest level of education?	Illiterate		Primary	Secondary	Tertiary	
What is the monthly income in your house hold?	<$50		$51-150	$151-250	>$250	
What is your occupation?	Home duties	Business selling	Farming	Government staff	NGO	Other
What is the occupation of the Father?	Home duties	Business selling	Farming	Government staff	NGO	Other
How many people live in your house?	.... people					
Does your house have running water?	Yes			No		
Does your house have electricity?	Yes			No		
What do you use to cook with?	Wood		Gas	Combination	other	

**Oral Health literacy**			
How important are baby teeth?			
Is there anything that you can do to prevent the primary teeth from breaking down with tooth decay?	Yes	No	Don’t Know
If yes then what can you do?	Brush teeth		Go to the dentist
	Use tooth paste		Avoid sweet food and drink
	Other:		
What do you think is the cause of dental caries on the teeth?	Sugar		Bacteria
	Inherited/genetic		Poor oral hygiene
	Other		
Did you notice whether your child has any white spots on their teeth?	Yes	No	Don’t know
How healthy do you think your child’s teeth will be when they go to school?	Likely to be healthy ←→ Likely to have a problem		
How likely do you think it will be that your child has a tooth ache in the next year?	High chance ←→ Low chance		
How important are the primary teeth?	Not so important		Very important

Nursing and feeding practices

	During the morning	During the middle of the day	During the afternoon	During the evening	During the night
How many times did you breastfeed yesterday?					
**How many times did your baby have the following things in a bottle yesterday?**					
Milk formula					
Rice Porridge					
Soya Milk					
Condensed milk					
Fruit juice or another sweet drink					
Water					
**How many times yesterday did your baby eat the following food:**					
Rice porridge					
Meat					
Fruits					
Egg					
Vegetable					
Khmer sweet cakes					
Packaged sweets					
Packaged snacks					

When did you or someone in your family first give your baby the following foods

	3 months	6 months	9 months	12 months	15 months	18 months	24 months
**Milk formula**							
**Rice Porridge**							
**Soya Milk**							
**Condensed milk**							
**Fruit juice or another sweet drink**							
**Water**							
**Rice porridge**							
**Meat**							
**Fruits**							
**Egg**							
**Vegetable**							
**Khmer sweet cakes**							
**Packaged sweets**							
**Packaged snacks**							

**Other Health problems:**	
How would you rate the over-all health of your child?	Excellent My child is often ill
Was your child born at full term or born early (pre-term)?	
During the last month has your child had any of the following	Chest infection/coughing/difficulty breathing
	Admitted to hospital
	Diarrhoea (more than 3 days)
	Taking any antibiotics?
What do you do when your child is upset?	Hug them or sooth them with words
	Play a game with them
	Give them something sweet to eat
	Other:

**Family Impact Score:** During the last 3 months, because of your child's teeth, lips, mouth or jaws, how often have you or another family member:					
Been upset?	Never ☐	Once or twice ☐	Sometimes ☐	Often ☐	Don't Know ☐
Felt guilty?	Never ☐	Once or twice ☐	Sometimes ☐	Often ☐	Don't Know ☐
Had your sleep disrupted?	Never ☐	Once or twice ☐	Sometimes ☐	Often ☐	Don't Know ☐
Taken time off work (e.g. due to pain, appointments, treatment)?	Never ☐	Once or twice ☐	Sometimes ☐	Often ☐	Don't Know ☐
Had less time for yourself or your family?	Never ☐	Once or twice ☐	Sometimes ☐	Often ☐	Don't Know ☐
Blamed yourself or another person in your family?	Never ☐	Once or twice ☐	Sometimes ☐	Often ☐	Don't Know ☐
Argued with your child or other members in your family?	Never ☐	Once or twice ☐	Sometimes ☐	Often ☐	Don't Know ☐
